# Investigating genetic overlaps of the genetic factor differentiating schizophrenia from bipolar disorder with cognitive function and hippocampal volume

**DOI:** 10.1192/bjo.2021.1086

**Published:** 2022-01-26

**Authors:** Kazutaka Ohi, Yukimasa Muto, Kentaro Takai, Shunsuke Sugiyama, Toshiki Shioiri

**Affiliations:** Department of Psychiatry, Gifu University Graduate School of Medicine, Japan; and Department of General Internal Medicine, Kanazawa Medical University, Japan; Department of Psychiatry, Gifu University Graduate School of Medicine, Japan

**Keywords:** Genetic correlation, schizophrenia, bipolar disorder, cognitive function, hippocampus

## Abstract

Schizophrenia and bipolar disorder display clinical similarities and dissimilarities. We investigated whether the genetic factor differentiating schizophrenia from bipolar disorder is genetically associated with cognitive phenotypes and hippocampal volumes. We revealed genetic overlaps of the genetic differentiating factor with low general cognitive ability, low childhood IQ, low educational attainment and reduced hippocampal volumes. The genetic correlations with low general cognitive ability and reduced hippocampal volumes were associated with risk of schizophrenia, whereas the genetic correlations with high childhood IQ and educational attainment were associated with risks of bipolar disorder. These findings suggest these disorders have disorder-specific genetic factors related to clinical phenotypes.

Schizophrenia and bipolar disorder are highly heritable disorders with clinical similarities and a complex, overlapping polygenic architecture.^1,2^ In contrast, a large-scale genome-wide association study (GWAS) identified two genome-wide significant loci differentiating schizophrenia from bipolar disorder.^3^ Although schizophrenia displays cognitive dysfunctions and reduced hippocampal volumes,^4^ there are somewhat limited data on these impairments in bipolar disorder. Genetic overlaps of risk for schizophrenia with cognitive impairments and reduced hippocampal volumes have been reported.^2,5,6^ These findings suggest that the two disorders would be distinct diagnoses, with disorder-specific genetic factors related to clinical phenotypes. However, it remains unknown whether a genetic factor differentiating schizophrenia from bipolar disorder can explain the dissimilarities in cognitive functions and hippocampal volumes. Here, we explored whether the genetic factor differentiating component is genetically associated with psychiatric disorders, cognitive phenotypes and hippocampal volumes.

## Method

To calculate genetic correlations attributable to genome-wide single nucleotide polymorphisms (SNPs) (polygenicity; many small genetic effects) between the genetic factor differentiating schizophrenia from bipolar disorder and psychiatric disorders, cognitive phenotypes and hippocampal volumes, we obtained GWAS summary statistics for the following: schizophrenia versus bipolar disorder,^3^ Psychiatric Genomics Consortium 2 (PGC2) for schizophrenia,^7^ PGC2 for bipolar disorder,^8^ major depression disorder (MDD), autism spectrum disorder (ASD), attention-deficit hyperactivity disorder (ADHD), general cognitive ability,^12^ childhood IQ, educational attainment and hippocampal volume.^15^ These data were extracted from the PGC (https://www.med.unc.edu/pgc/results-and-downloads), the Centre for Cognitive Ageing and Cognitive Epidemiology at the University of Edinburgh (http://www.ccace.ed.ac.uk/node/335), the Social Science Genetic Association Consortium (https://www.thessgac.org/data) and the Enhancing Neuroimaging Genetics through Meta-analysis (ENIGMA2; http://enigma.ini.usc.edu/research/download-enigma-gwas-results/) (Table 1).

**Table 1:** Demographic information of each genome-wide association study

			Sample sizes			
		Genome-wide significant loci	Total	Cases	Controls^a^	Single nucleotide polymorphisms, *n*
Schizophrenia versus bipolar disorder	Ruderfer et al (2018)^3^	2	38 855	23 585^b^	15 270^c^	1 064 728
Schizophrenia	Ripke et al (2014)^7^	108	82 315	35 476	46 839	1 125 108
Bipolar disorder	Stahl et al (2019)^8^	30	51 710	20 352	31 358	1 110 247
Major depression disorder	Wray et al (2018)^9^	44	173 005	59 851	113 154	1 110 699
Autism spectrum disorder	Grove et al (2019)^10^	5	46 350	18 381	27 969	1 050 225
Attention-deficit hyperactivity disorder	Demontis et al (2019)^11^	12	55 374	20 183	35 191	1 069 649
General cognitive ability	Davies et al (2018)^12^	148	282 014	−	−	1 204 339
Childhood IQ	Benyamin et al (2014)^13^	0	12 441	−	−	804 043
Educational attainment	Lee et al (2018)^14^	1271	1 131 881	−	−	1 177 103
Hippocampal volume	Hibar et al (2015)^15^	2	13 163	−	−	1 161 732

a. Controls or pseudocontrols from family trio samples.

b. Schizophrenia.

c. Bipolar disorder.

Linkage disequilibrium score regression (LDSC) analysis can estimate the genetic SNP correlations (*r_g_*) from GWASs.^5,16,17^ For each GWAS, an LDSC was carried out by regressing the GWAS test statistics (*χ^2^*) onto each SNP's linkage disequilibrium score. Genetic correlations were calculated by LDSC. This study was approved by each local ethical committee of the relevant institutions. Informed consent was obtained from all participants and/or their families in each study cohort. The detailed information in each GWAS and LDSC analysis have been described previously, and are briefly summarised in the Supplementary Material available at https://doi.org/10.1192/bjo.2021.1086.

## Results

**Fig. 1:**
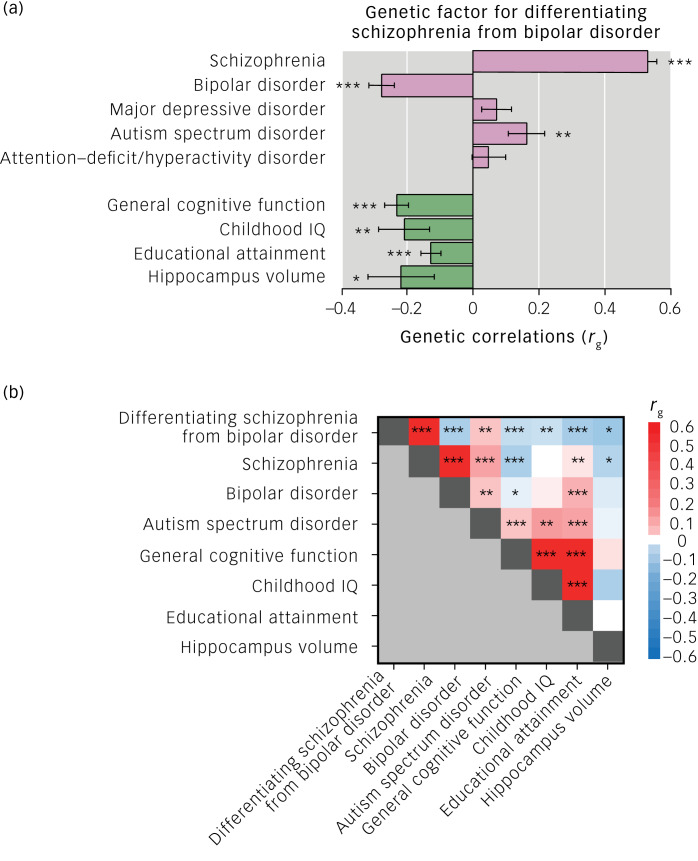
(a) Genetic correlations (*r_g_*) of genetic factor differentiating schizophrenia from bipolar disorder with psychiatric disorders, cognitive functions and hippocampal volumes. Error bars show s.e. of the *r_g_*. (b) Genetic correlations (*r_g_*) across genome-wide association study results. The colour scale represents the *r_g_* values. Genetic correlations were estimated with linkage disequilibrium score regression. *P < 0.05, **P < 0.01, ***P < 0.001.

As expected, the genetic component differentiating schizophrenia from bipolar disorder was positively correlated with the risk of schizophrenia (*r_g_* ± s.e. = 0.53 ± 0.03, *P* = 1.21 × 10^−82^), and negatively correlated with the risk of bipolar disorder (*r_g_* ± s.e. = −0.28 ± 0.04, *P* = 5.04 × 10^−13^) (Fig. 1(a)). Among other psychiatric disorders, there was positive genetic correlation between the differentiating genetic factor and ASD (*r_g_* ± s.e. = 0.16 ± 0.05, *P* = 2.90 × 10^−3^). There were no significant genetic correlations of the differentiating factor with MDD or ADHD (*P* > 0.05). The genetic factor differentiating schizophrenia from bipolar disorder was genetically negatively correlated with all examined cognitive phenotypes and hippocampal volumes (Fig. 1(a); general cognitive function (*r_g_* ± s.e. = −0.23 ± 0.04, *P* = 6.80 × 10^−11^), childhood IQ (*r_g_* ± s.e. = −0.21 ± 0.08, *P* = 7.30 × 10^−3^), educational attainment (*r_g_* ± s.e. = −0.13 ± 0.03, *P* = 2.66 × 10^−5^) and hippocampal volume (*r_g_* ± s.e. = −0.22 ± 0.10, *P* = 0.031)). As shown in genetic correlations across phenotypes (Fig. 1(b)), genetic correlations of the genetic differentiating factor with general cognitive function and hippocampal volume would be derived from genetic correlations of schizophrenia with these phenotypes. In contrast, genetic correlations of the genetic differentiating feature with childhood IQ and educational attainment would be derived from genetic correlations of bipolar disorder and/or ASD with these phenotypes.

## Discussion

We revealed genetic overlaps of the genetic variants differentiating schizophrenia from bipolar disorder with risk of ASD, low general cognitive ability, low childhood IQ, low educational attainment and reduced hippocampal volumes. These genetic overlaps may be attributed to genetic risks for schizophrenia or bipolar disorder, or both. We further found that the genetic correlations of the genetic factor differentiating schizophrenia from bipolar disorder with low general cognitive ability and reduced hippocampal volumes are associated with risk of schizophrenia, and the genetic correlations of the genetic factor differentiating bipolar disorder from schizophrenia with high childhood IQ and educational attainment are associated with risks of bipolar disorder and/or ASD. The disorder-specific genetic liability could contribute to the clinical dissimilarities between schizophrenia and bipolar disorder. Current schizophrenia diagnoses may aggregate at least two subtypes:^18^ patients who resemble high intelligence and bipolar disorder (similarities), and patients who show cognitive impairments that are independent of bipolar disorder (dissimilarities). However, it remained unclear whether low intelligence causes schizophrenia or schizophrenia causes intelligence decline. Using summary data-based Mendelian randomisation,^19^ we recently demonstrated that low intelligence was bidirectionally associated with a high risk of schizophrenia, whereas the schizophrenia-specific genetic factors might be mainly affected by impairment in premorbid intelligence.^20^ Future study is required to reveal causal association between reduced hippocampal volumes and risk of schizophrenia.

Interestingly, there were no significant correlations between the genetic factor differentiating schizophrenia from bipolar disorder and MDD or ADHD. Comparing genetic correlations between schizophrenia and MDD with those between bipolar disorder and MDD, and genetic correlations between schizophrenia and ADHD with those between bipolar disorder and ADHD, both schizophrenia and bipolar disorder correlations with MDD and ADHD were similar.^2^ The absence of MDD or ADHD correlations with the differentiating factor might reflect similar degrees of these genetic correlations with schizophrenia and bipolar disorder.

Our findings suggest that cognitive impairments and reduced hippocampal volumes could genetically distinguish schizophrenia from bipolar disorder, and may be useful for improving diagnosis and treatment.

## Data Availability

The data that support the findings of this study are available from the corresponding author, K.O., on reasonable request.

## Supplementary material

For supplementary material accompanying this paper visit http://doi.org/10.1192/bjo.2021.1086. click here to view supplementary material
