# Supplementary material for: Investigating genetic overlaps of the genetic factor differentiating schizophrenia from bipolar disorder with cognitive function and hippocampal volume
Source: BJPsych Open. 2022 Jan 26;8(1):e33. doi: 10.1192/bjo.2021.1086 (PMC8811788; doi:10.1192/bjo.2021.1086)
Supplement: Supplementary file 1 [file S2056472421010863sup001.docx]

**Supplementary Information**

**Supplementary Methods**

To calculate genetic correlations attributable to genome-wide SNPs (polygenicity; many small genetic effects) between genetic factor for differentiating SCZ from BIP with psychiatric disorders, cognitive phenotypes and hippocampal volumes, GWAS summary statistics regarding SCZ *vs* BIP,^1^ Psychiatric Genomics Consortium (PGC)2 for SCZ,^2^ PGC2 for BIP,^3^ MDD,^4^ ASD,^5^ ADHD,^6^ general cognitive ability,^7^ childhood IQ,^8^ educational attainment,^9^ and hippocampal volume^10^ from the PGC, the Centre for Cognitive Ageing and Cognitive Epidemiology at the University of Edinburgh (CCACE), the Social Science Genetic Association Consortium (SSGAC), and the Enhancing Neuroimaging Genetics through Meta-analysis (ENIGMA2) study were extracted from public databases (PGC, <https://www.med.unc.edu/pgc/results-and-downloads>; CCACE, <http://www.ccace.ed.ac.uk/node/335>; SSGAC, <https://www.thessgac.org/data>; ENIGMA, <http://enigma.ini.usc.edu/research/download-enigma-gwas-results/>). This study was approved by each local ethical committee of relevant institutions. Informed consent was obtained from all participants and/or their families in each study cohort. The detailed information in each GWAS have been described previously,^11-13^ and briefly summarized in as follows.

**SCZ *vs* BIP**

The samples from the SCZ and BIP Working Group of the PGC consisted of 23,585 SCZ cases and 15,270 BIP cases of European ancestry.^1^ Quality control (QC) and imputation were performed on each of the study cohort datasets, according to standards established by the PGC.^2^ Genotype imputation was performed using the 1000 Genomes Project Phase 3 dataset as a reference panel. SNPs with minor allele frequency (MAF) >0.01 and imputation quality (INFO) >0.3 were retained.

**SCZ**

The samples from the SCZ Working Group of the PGC2 consisted of 32,405 SCZ cases and 42,221 controls of European ancestry, 1,235 trio samples of European ancestry, 1,836 SCZ cases and 3,383 controls from East Asia.^2^ These samples were genotyped using different arrays for each study site. QC and imputation were performed for each dataset separately. The applied QC parameters for retaining SNPs and subjects were as follows: SNP missingness <0.05 (before sample removal); subject missingness <0.02; deviation from autosomal heterozygosity (|F_het_| <0.2); SNP missingness <0.02 (after sample removal); difference in SNP missingness between cases and controls of <0.02; and deviation from Hardy-Weinberg equilibrium (HWE) (*p*<1.0×10^-6^ in controls or *p*<1.0×10^-10^ in cases). Genotype imputation was performed using the 1000 Genomes Project Phase 1 dataset as a reference panel. SNPs with imputation quality (INFO>0.6) were retained.

**BIP**

The samples from the BIP Working Group of the PGC2 comprised 20,352 BIP cases and 31,358 controls of European descent.^3^ These samples were genotyped using different arrays for each site. Individual genotype data were processed using the PGC pipeline^2^ for standardized QC, imputation, and analysis. The applied QC parameters were the same as mentioned above. Genotype imputation was performed using the 1000 Genomes Project Phase 3 dataset as a reference panel. SNPs with imputation quality (INFO ≥0.3) and MAF ≥0.01 were retained.

**MDD**

The samples from the MDD Working Group of the PGC comprised 59,851 MDD cases and 113,154 controls of European descent.^4^ These samples were genotyped using different arrays for each study site. QC and imputation were performed for each dataset separately. QC were performed according to standards from the PGC^2^ or the similar pipeline. Genotype imputation was performed using the 1000 Genomes Project Phase 1 dataset as a reference panel.

**ASD**

The samples from the PGC ASD Working Group and the iPSYCH comprised 18,381 ASD cases and 27,969 controls of European descent.^5^ These samples were genotyped using different arrays for each study site. QC and imputation were performed for each dataset separately. Ricopili,^2^ the pipeline developed by the PGC, was used for QC and imputation. Genotype imputation was performed using the 1000 Genomes Project Phase 3 dataset as a reference panel.

**ADHD**

The samples from the PGC ADHD Working Group and the iPSYCH comprised 20,183 ADHD cases and 35,191 controls of mainly European descent.^6^ QC procedures were performed on the genotyped markers and individuals in each cohort using a standardized pipeline.^2^ Genotype imputation was performed using the 1000 Genomes Project Phase 3 dataset as a reference panel.

**General cognitive ability**

The samples comprised a population-based set of 282,014 individuals of European ancestry from the Cohorts for Heart and Aging Research in Genomic Epidemiology (CHARGE) and Cognitive Genomics Consortium (COGENT) consortia and UK Biobank.^7^ All individuals were aged between 16 and 102 years. Individuals with clinical stroke or prevalent dementia were excluded. General cognitive ability is measured as a general cognitive function component constructed from a number of cognitive tasks in the cohorts of the CHARGE and COGENT consortia. General cognitive ability in the UKB is assessed by verbal and numerical reasoning (VNR: ‘fluid’ cognitive test) consisting of 13 multiple-choice questions. These samples were genotyped using different arrays for each study site. QC procedures were performed on the genotyped markers and individuals in each cohort. Genotype imputation was performed using the 1000 Genomes Project or the Haplotype Reference Consortium (HRC) dataset as a reference panel.

**Childhood IQ**

The samples from the Childhood Intelligence Consortium (CHIC) comprised 12,441 children of European ancestry.^8^ All children were aged between 6 and 18 years. The childhood IQ is assessed using the best available measure of general cognitive ability (*g*) or IQ, derived from diverse tests that assess both verbal and non-verbal ability. These samples were genotyped using different arrays for each study site. QC procedures were performed on the genotyped markers and individuals in each cohort. Genotype imputation was performed using the HAPMAP II CEU dataset as a reference panel.

**Educational attainment**

The samples comprised 1,131,881 individuals of European ancestry.^9^ Educational attainment is measured as the number of years of schooling that individuals completed. Educational attainment was measured at an age of at least 30. These samples were genotyped using different arrays for each study site. A uniform set of QC procedures was performed on the genotyped markers and individuals in each cohort. Genotype imputation was performed using the 1000 Genomes Project Phase 3 dataset as a reference panel.

**Hippocampal volume**

The samples from the ENIGMA consortium consisted of 13,688 participants of European ancestry.^10^ Brain scans were processed at each site locally and the hippocampal volumes were extracted, following a standardized protocol procedure (<http://enigma.ini.usc.edu/protocols/imaging-protocols/>). These samples were genotyped using different arrays for each study site. QC filtering was applied to remove genotyped SNPs with low MAF (<0.01), poor genotype call rate (<0.95), and deviations from HWE (*p*<1.0×10^-6^). Genotype imputation was performed using the 1000 Genomes Project Phase 1 dataset as a reference panel.

**Linkage Disequilibrium Score regression analysis**

Linkage Disequilibrium Score regression (LDSC) analysis can estimate the genetic SNP correlations (*r_g_*) from GWASs.^11, 13, 14^ Regression weights (LD scores, ‘eur_w_ld_chr/’ files <https://github.com/bulik/ldsc>) were precomputed using the European-ancestry samples of the 1000 Genomes Project. To restrict the analysis to well-imputed SNPs, the imputed and directly genotyped SNPs in each GWAS were filtered to SNPs that overlapped with a HapMap3 SNP panel. Only SNPs with an imputation INFO score>0.90 and MAF>0.01 were included, and insertion-deletion polymorphisms (indels), structural variants, strand-ambiguous SNPs and SNPs with extremely large effect sizes were excluded. For each GWAS, an LD regression was carried out by regressing the GWAS test statistics (*χ^2^*) onto each SNP’s LD score. Using the LDSC, we calculated the genetic correlation among phenotypes.

**Supplementary References**

**1.** Ruderfer D, Ripke S, McQuillin A, et al. Genomic Dissection of Bipolar Disorder and Schizophrenia, Including 28 Subphenotypes. *Cell.* 2018;173(7):1705-1715 e1716.

**2.** Ripke S, Neale BM, Corvin A, et al. Biological insights from 108 schizophrenia-associated genetic loci. *Nature.* 2014;511(7510):421-427.

**3.** Stahl EA, Breen G, Forstner AJ, et al. Genome-wide association study identifies 30 loci associated with bipolar disorder. *Nat Genet.* 2019;51(5):793-803.

**4.** Wray NR, Ripke S, Mattheisen M, et al. Genome-wide association analyses identify 44 risk variants and refine the genetic architecture of major depression. *Nat Genet.* 2018;50(5):668-681.

**5.** Grove J, Ripke S, Als TD, et al. Identification of common genetic risk variants for autism spectrum disorder. *Nat Genet.* 2019;51(3):431-444.

**6.** Demontis D, Walters RK, Martin J, et al. Discovery of the first genome-wide significant risk loci for attention deficit/hyperactivity disorder. *Nat Genet.* 2019;51(1):63-75.

**7.** Davies G, Lam M, Harris SE, et al. Study of 300,486 individuals identifies 148 independent genetic loci influencing general cognitive function. *Nat Commun.* 2018;9(1):2098.

**8.** Benyamin B, Pourcain B, Davis OS, et al. Childhood intelligence is heritable, highly polygenic and associated with FNBP1L. *Mol Psychiatry.* 2014;19(2):253-258.

**9.** Lee JJ, Wedow R, Okbay A, et al. Gene discovery and polygenic prediction from a genome-wide association study of educational attainment in 1.1 million individuals. *Nat Genet.* 2018;50(8):1112-1121.

**10.** Hibar DP, Stein JL, Renteria ME, et al. Common genetic variants influence human subcortical brain structures. *Nature.* 2015;520(7546):224-229.

**11.** Ohi K, Shimada T, Kataoka Y, et al. Genetic correlations between subcortical brain volumes and psychiatric disorders. *Br J Psychiatry.* 2020;216(5):280-283.

**12.** Ohi K, Nishizawa D, Shimada T, et al. Polygenetic Risk Scores for Major Psychiatric Disorders Among Schizophrenia Patients, Their First-Degree Relatives, and Healthy Participants. *Int J Neuropsychopharmacol.* 2020;23(3):157-164.

**13.** Ohi K, Otowa T, Shimada M, Sasaki T, Tanii H. Shared genetic etiology between anxiety disorders and psychiatric and related intermediate phenotypes. *Psychol Med.* 2020;50(4):692-704.

**14.** Bulik-Sullivan BK, Loh PR, Finucane HK, et al. LD Score regression distinguishes confounding from polygenicity in genome-wide association studies. *Nat Genet.* 2015;47(3):291-295.
